# A comparative study of glaucoma referrals in Southeast Scotland: effect of the new general ophthalmic service contract, Eyecare integration pilot programme and NICE guidelines

**DOI:** 10.1186/s12886-015-0161-5

**Published:** 2015-12-07

**Authors:** Karim El-Assal, Jonathan Foulds, Stuart Dobson, Roshini Sanders

**Affiliations:** Department of Ophthalmology, Queen Margaret Hospital, Whitefield Road, KY12 0SU Dunfermline, Fife UK; Department of Ophthalmology, Ninewells Hospital, Dundee, UK; Medical Informatics, Queen Margaret Hospital, Dunfermline, Fife UK

**Keywords:** Glaucoma, Optometrist, Referral, Applanation tonometry, Visual fields, Fundoscopy

## Abstract

**Background:**

Glaucoma is a progressive disease responsible for the second commonest cause of blindness in the UK. Identifying appropriate patients for hospital care remains an ongoing challenge for all UK hospital glaucoma services. The purpose of our study is to evaluate accuracy and outcome of community optometry referrals before and after implementation of the new general ophthalmic service contract in 2006, the Eyecare Integration Programme pilot in 2008 and the effect of NICE guidelines in glaucoma in 2009, over a 12-year period

**Methods:**

A retrospective case analysis using a glaucoma electronic patient record was performed encompassing two six-year periods, 2000–2006 (Group A), and 2007–2012 (Group B).

**Results:**

One thousand six hundred twenty-two new patients’ records were analysed. Waiting times reduced from 12.3 to 9.4 weeks. Significantly more patients kept first appointment (*p* = 0.0002) in group B. Glaucoma symptoms were significantly more in group A (*p* <0.0001) and only three patients lost Snellen’ visual acuity before appointment in group B compared to 12 in group A. Documentation of intraocular pressure was made in 74.1 % of Group A and 75.9 % of Group B, optic disc appearance in 85.4 % of Group A, and 93 % of Group B and visual fields in 84.4 % of Group A and 81.3 % of Group B. Significantly less normal (*p* < 0,0001), more glaucoma suspects (*p* < 0.0001), more open angle glaucoma (*p* = 0.0006) and fewer other conditions (*p* = 0.0024) were present in group B, compared to group A.

**Conclusion:**

Patients were referred earlier with shorter waiting times for hospital appointments with the new Scottish general ophthalmic service and Eyecare Integration Programme. Additionally there were fewer false positive referrals with more diagnosis of glaucomatous disease. We discuss the benefits of these national screening and referral pathways together with their limitations and further refinements.

## Background

Glaucoma is a progressive disease responsible for the second commonest cause of blindness in the UK [1]. Early detection and treatment is key to minimising visual loss. Glaucoma suspect referrals account for at least 20 % of new referrals to the Hospital Eye Services (HES) and an even larger percentage of return visits [2].

Identifying appropriate patients for hospital care remains an ongoing challenge for all UK HES glaucoma services [3]. Refining patient referral information to the HES has the potential to identify urgent cases early and reduce the false positive referral rate. Studies have shown that the positive predictive value of glaucoma referrals from optometrists is improved when all three glaucoma screening tests: intraocular pressure measurement (IOP), visual field and optic disc assessment, are deployed [4]. The pressure on HES glaucoma services has created the need for many departments to introduce innovative ways of service delivery to include shared care models and virtual glaucoma clinics [5, 6].

In 2006, a new General Ophthalmic Services (GOS) contract was implemented by the Scottish government, with a mandate to perform contact tonometry, dilated fundoscopy and automated perimetry in all patients with suspected glaucoma [7]. The new GOS contract was introduced as a step change in the delivery of community eye care in Scotland and had two components: the primary and supplementary examinations, which makes it possible for optometrists to refine referral with repeat testing of patients before referral to secondary care as this has shown substantial benefit [8]. The eye test is universally free and this service structure is unique to Scotland within a national public health service.

In 2008, as part of a pilot study for the Eyecare Integration Programme (EIP) Scotland, the region of Fife redesigned services to incorporate electronic referrals from community optometry to HES [9, 10]. This pathway allowed optometrists to refer electronically using NHS mail. The referral also allowed for attachments of optic disc images and visual fields. In 2009 the Scottish government offered community grants for the purchase of digital cameras to all Scottish community optometrists [9, 10].

In 2009 NICE guidelines for glaucoma were issued and one of the recommendations was that an IOP above 21 mmHg merited referral to HES regardless of all other findings [11]. This was potentially set to increase the referral rate from community to HES, regardless of any regional service infrastructure processes.

Our study evaluates the quality of 1622 referrals from optometrists to HES before and after implementation of the new GOS contract, EIP pilot and NICE guidelines over a twelve year period

## Methods

Fife is a region in southeast Scotland with three hospitals serving a population of 450,000 [12]. This study electronically examined all new glaucoma patient details of those referred to Queen Margaret Hospital, Dunfermline over twelve years from 2000 till 2012. The midpoint of this study is 2006 when the new GOS was introduced. There was a gap of six months in 2006 when new patient details were not entered electronically due to changes in the patient electronic management system, which also coincided with introduction of the new GOS contract

The glaucoma electronic patient record (GEPR) was designed within the hospital electronic patient management system Oasis (© 2013 Oasis Medical Solutions Limited- London) [13]. The record was divided in three parts to include:

1. Optometry referral information
2. Hospital medical history and examination details
3. Patient outcome (diagnosis).

The GEPR details and questions designed were based on previous work published by the department on causes and recommendations for glaucoma patients with visual impairment [14, 15].

Ethical approval was sought from the Fife Research Ethics Committee. Since all the data was anonymised and the data was an audit of clinical parameters the committee decided that full approval was not required and we were given permission to progress with our study.

The service in Fife is designed so that all new referrals for glaucoma are seen in a dedicated glaucoma service run by a trained glaucoma team consisting of consultant, associate specialist, senior glaucoma trainee and two specialist accredited glaucoma nurses. All new diagnoses were discussed with the consultant and any cases with complex diagnosis were seen only by the consultant. Protocols are in place for consistent and mandatory methods of history and examination. All new patients have a full ophthalmic examination to include slit lamp examination, Goldman contact tonometry, gonioscopy, dilated fundoscopy, photography and Humphrey Sita Standard 24–2 perimetry. Corneal pachymetry findings have been excluded as its use became consistent only half way through the study and therefore was not a comparable finding across the study period. Likewise Optical Coherence Tomography was used in selected cases and therefore the findings did not allow for meaningful and consistent comparison across the study group. All outcomes were based on comparison of findings at first hospital visit to that of the referring details from community optometrist. All referrals had an optometry report. Acute glaucoma cases were seen on the day of referral at the emergency eye services and not included in this series. These were typically cases of acute angle closure glaucoma and also acute secondary glaucoma.

The highest optometry IOP at referral was documented with the vast majority using contact tonometry and less than 10 % using non-contact tonometry. Optometry disc findings were considered abnormal if the cup disc ratio was above 0.4 as this is known to occur in less than 10 % of the normal population [16] (Scottish optometrists were trained with the Armaly system of disc analysis to have a high index of glaucoma suspicion in these cases) or had other signs of glaucoma such as pallor, notching or disc haemorrhage. Optometry visual field testing was performed using Humphrey, FDT and Hensons technologies in 78 % and a mixture of other screening technologies to include Friedmont, Medmont abd Dicon strategies in the remainder. Patients were categorized according to the clinical findings on the first appointment at HES as:

1. Normal examination- no IOP, disc or visual field finding consistent with glaucoma or ocular hypertension
2. Ocular hypertension - IOP > 21mmhg but no disc or visual fields suggestive of glaucoma
3. Glaucoma suspect - glaucoma could not be ruled out or confirmed as a result of unclear visual field or disc findings and required further testing
4. Low tension glaucoma - evidence of glaucomatous damage in the form of optic disc or visual field findings and IOP ≤ 22mmhg
5. Open angle glaucoma - IOP, disc appearance and visual fields consistent with glaucoma with open angles on gonioscopy
6. Other diagnosis - patients did not conform to any of the above at the first visit and required further testing. In particular patients with narrow angles or shallow anterior chambers were mostly brought back to a separate laser clinic for reassessment and treatment before a definitive diagnosis was made.

Patients were divided into two groups from 2000 to 2006 (group A) and from 2007 to 2012 (group B). All collected data was compared between the two groups. Details from the GEPR were retrieved electronically using Business Objects software (SAP® -Germany) and transported to an excel spread sheet. Data was analysed using comparison of proportions (chi squared test) with a *p* value of ≤0.05 taken to indicate statistical significance.

## Results

The Queen Margaret Hospital saw 1622 glaucoma referrals over the set 12 year period. Group A (June 2000 – May 2006, 72 months) included 835 patients while group B (January 2007 – December 2012, 72 months) included 787 patients.

The average age at presentation was 64 years for group A (range 35–96) and 66 for group B (range 41–94). Four hundred and three patients were male (48.3 %) and 432 female (51.7 %) in group A with 380 (48.3 %) male and 407 female (51.7 %) in group B.

In group A, 698 patients (83.6 %) were referred to the glaucoma clinic directly by the optometrist, 40 patients (4.8 %) by the general practitioner (GP) and 97 (11.6 %) by other sources (such as other medical specialities and NHS 24). In group B, 642 patients (81.6 %) were referred directly by the optometrist, 34 patients (4.3 %) by the GP and 111 (14.1 %) by other sources. Patients referred by sources other than optometry were directed to have a local optometry examination before hospital appointment so that all patients had an optometry report (Table 1).

**Table 1:** Attendance and sources of referral in the two groups

	Group A (835 patients)	Group B (787 patients)
DNA ^a^ first appointment	49 (5.9 %)	17 (2.2 %)
Referred by Optometrist	698 (83.6 %)	642 (81.6 %)
Referred by GP	40 (4.8 %)	34 (4.3 %)
Other	97 (11.6 %)	111(14.1 %)
Referral to clinic time	86 days	66 days

^a^Did not attend

Patients in group A took an average of 86 days (12.3 weeks) from referral to clinic visit and this was 66 days (9.4 weeks) for group B. Patients that did not attend (DNA) the first appointment were significantly more (Χ^2^ = 14.27, *p* = 0.0002) in group A (49, 5.9 %) than in group B (17, 2.2 %), (Table 1).

Typical symptoms associated with glaucoma such as awareness of a blind spot or field defect, altered or gradual reduction in vision or episodes of blurring and pain that may indicate intermittent angle closure were documented in the optometrist referral in 586 patients (70.2 %) in group A and in 425 patients (54 %) in group B There were significantly more patients with glaucoma symptoms in group A compared to group B (Χ^2^ = 45.2, *p* < 0.0001) (Table 2). Loss of one line of Snellen’s visual acuity attributable to purely delay in appointment between community examination and hospital appointment occurred in 12 patients in group A and 3 patients in group B (Table 2). Further verification that visual acuity loss was from untreated glaucoma and not other diseases was made as these cases were individually studied and reported at monthly glaucoma clinical governance meetings.

**Table 2:** Documentation of findings by referring optometrist

	Group A (835 patients)	Group B (787 patients)
Glaucoma symptoms	586 (70.2 %)	425 (54 %)
Positive family history	276 (33.1 %)	190 (24.2 %)
IOP^a^	619 (74.1 %)	597 (75.9 %)
Optic disc appearance	713 (85.4 %)	614 (78 %)
Optic disc images	6 (0.7 %)	545 (69 %)
Visual field	705 (84.4 %)	640 (81.3 %)

^a^Intra-ocular pressure

A positive family history of glaucoma was documented in the referral in 276 patients (33.1 %) in group A and in 190 patients (24.1 %) in group B. At the HES, 208 (24.9 %) patients in group A and 215 (27.3 %) patients in group B were found to have a positive family history of glaucoma.

The referring optometrist documented the IOP in 618 patients (74 %) in group A, and in 597 referrals (75.9 %) in group B. The average optometry referral IOP was 23.4 in group A and 21.4 in group B. These figures were 20.6 in group A (815 patients, 97.6 %) and 20.1 in group B (768, 97.6 %) at the HES.

The optometrist documented visual fields in 705 (84.4 %) patients in group A and 640 (81.3 %) patients in group B. In group A the optometrist found 240 (28.7 %) normal and 465 (55.7 %) abnormal visual fields in one or both eyes. In group B the optometrist found 241 (30.6 %) normal and 399 (50.7 %) abnormal visual fields in one or both eyes. In the HES 785 (94 %) patients in group A and 748 (95 %) in group B had visual field testing (Table 2).

In group A, 713 patients (85.4 %) had optometry documentation of one or both optic discs. In group B 614 (78 %) patients had documentation of disc appearances and 545 (69 %) patients had fundus disc images. In total 732 patients (93 %) had referral information on optic discs in group B. At HES 785 (94 %) patients in group A and 748 (95 %) patients in group B had fundus examination.

For the purposes of whole group comparison between optometry and HES disc findings, group A was defined as having 1670 discs and group B as having 1574 discs. In group A the optometrist deemed 538 (32.2 %) discs to be normal and this compared to 872 (52.2 %) in HES. In group B the optometrist deemed 436 (27.7 %) discs to be normal and this compared to 625 (39.7 %) in the HES. In group A the optometrist deemed 640 (38.3 %) discs as abnormal and this compared to 675 (40.4 %) in the HES. In group B the optometrist deemed 687 (43.6 %) discs as abnormal and this compared to 949 (60.3 %) at HES. There were significantly fewer normal (Χ^2^ = 51.0, *p* < 0.0001) and more abnormal (Χ^2^ = 77.89, *p* < 0.0001) disc findings in HES in group B than group A. (Table 3)

**Table 3:** Optic disc appearance findings by optometrists and HES in both groups

	Group A (1670 eyes)		Group B (1574 eyes)	
	Normal	Abnormal	Normal	Abnormal
Optom^a^	538 (32.2 %)	640 (38.3 %)	436 (27.7 %)	687 (43.6 %)
HES^b^	872 (52.2 %)	675 (40.4 %)	625 (39.7 %)	880 (55.9 %)

^a^Optometrist

^b^Hospital eye service

**Table 4:** Diagnosis at first HES^a^ appointment in both groups

	Group A (1670 eyes)	Group B (1574 eyes)	Statistical significance
Normal	633	380	Χ^2^ = 71.4,
	37.9 %	24.1 %	*p* < 0.0001
Glaucoma suspect	425	659	Χ^2^ = 98.2,
	25.4 %	41.9 %	*p* < 0.0001
Ocular hypertension	286	242	Χ^2^ = 1.82,
	17.1 %	15.4 %	*p* = 0.177
Low tension glaucoma	16	12	Χ^2^ = 0.36,
	0.96 %	0.76 %	*p* = 0.547
Open angle glaucoma	73	113	Χ^2^ = 11.82
	4.4 %	7.2 %	*P* = 0.0006
Awaiting test	237	168	Χ^2^ = 9.18
	14.2 %	10.7 %	*p* = 0.0024

^a^Hospital eye service

Hospital diagnosis was made on individual eyes, thus a total of 1670 in group A and 1574 in group B (Table 4). At the first hospital appointment 633 eyes (37.6 %) were found to be normal in group A compared to 380 eyes (24.1 %) in group B. The initial working diagnosis in group A was glaucoma suspect in 425 eyes (25.4 %), ocular hypertension in 286 eyes (17.3 %), normal tension glaucoma in 16 eyes (1 %), chronic open angle glaucoma in 73 eyes (4.4 %), and awaiting further tests and other conditions in 237 eyes (14.2 %) .In group B, 659 eyes (41.9 %) were diagnosed initially as glaucoma suspect, 242 eyes (15.4 %) as ocular hypertension, 12 eyes (0.7 %) as normal tension glaucoma, 113 eyes (7.2 %) as chronic open angle glaucoma, and awaiting further tests and other conditions in 164 eyes (10.5 %).

There were significantly fewer normal patients (*p* < 0,0001), more glaucoma suspects (*p* < 0.0001), more open angle glaucoma patients (*p* = 0.0006) and fewer other conditions (*p* = 0.0024) in group B, compared to group A.

## Discussion

There have been a plethora of UK referral schemes described in the literature in the last few years [17, 18] with the largest and most comparable series to our study by Bowling et al. who reported on 2505 referrals between 1994 and 2004 in the Oxford region [19]. Since this study to our knowledge this is the second largest series of new glaucoma referrals to be analysed within the UK NHS system and only the second analysis in Scotland (preceded by the Grampian study in 2006 by Ang et al. [20]).

In the course of our study the significant changes in Scotland were the introduction of the GOS contract in 2006, the pilot EIP in 2008 and the NICE guidelines for glaucoma in 2009 [7, 10, 11]. As the Association of Optometrists endorsed nationwide application of NICE, there was compulsion for Scottish optometrists to conform regardless of any local Scottish arrangements. The aim of our study was to examine the pattern of referrals before and after the introduction of the above changes. We acknowledge that breaking down the study periods to 2006 – 2008, 2008 – 2009 and post NICE after 2009 may have given more meaningful referral trends. However this would have led to multiple small cohorts of data that may not have resulted in meaningful interpretation, hence our decision to have two study periods

Both groups were similar in age and gender and this is as expected as Fife is considered to have a geographically stable population [12]. The waiting time for hospital appointments was reduced in group B (12.3 to 9.4 weeks) almost singularly due to increasing electronic referral in the second half of the study. The delay for appointment in the first half of the study was due to the optometry referral being posted to the GP who then posted the referral with medical history to the hospital. Instead of the lengthy paper route of referrals from optometry to general GP to hospital, from 2008 direct electronic referral into the ophthalmology department with on the day vetting (the referral being seen and allocated appointment on the same day) led to faster appointment processes. Concomitantly patient medical history was sent electronically from GP practice to hospital, but did not hold up either the vetting or hospital appointment processes [9]. Capacity within the glaucoma service was unchanged over the study period so that an average of nine weeks was the “best” wait for a new glaucoma patient.

Patients were significantly more likely (*p* = 0.0002) to keep their first hospital appointment in the second half of the study. This may be the result of reduced waiting times thus making patients less likely to forget their appointments. It may also reflect better information and patient education at the optometry examination, about the possibility of having glaucoma and the importance of keeping their HES appointments. Studies have shown that delay in the first appointment has the potential to have a significant impact on visual outcome and untreated open angle glaucoma can cause registerable blindness within three years of onset [13, 21]. We acknowledge that socioeconomic status and co-morbidities also influence patient compliance with appointments.

It is hugely encouraging that significantly fewer patients had glaucoma symptoms and loss of Snellen’s visual acuity in group B compared to group A (*p* < 0.0001). This alludes to patients being screened and referred earlier in the glaucomatous process in the primary care setting. This is crucial progress in the primary and secondary interface of glaucoma care that minimises final glaucomatous visual loss.

Consistently, IOP was measured in 75 % of patients referred to the glaucoma service in both groups while this figure was 97 % in the HES. It is conceivable that some of the explanation is genuine difficulty with IOP measurement in the community as there are some limitations with both equipment consistency and clinical skill. However the failure to document IOP in nearly a quarter of patients seems excessive and community optometrists should strive towards IOP measurement in every patient referred to the glaucoma service.

Optometry disc examination was completed in 85.4 % in group A and a combination of optic disc examination and optic disc images resulted in 93 % of patients having optic disc information in group B. This is nearly the same as in hospital where 95 % had optic disc information (5 % had attempted fundus examination but no definitive findings due to presence of cataract or non- compliance with examination). This is another important progressive step in community screening. Measuring IOP and carrying out visual field testing are technical skills. However disc interpretation requires good clinical skills and it is the most challenging part of the glaucoma screening process prone to misinterpretation and failure to diagnose pathology. Having the ability to send optic disc images not only compensates for this variable clinical skill but arguably gives more accurate information than disc interpretation and at the hospital end significantly improves the quality of referral information and vetting outcome.

The importance of good clinical disc interpretation is further substantiated by the fact that group B had significantly fewer normal and more abnormal disc findings in HES (*p* <0.0001). This is mirrored also by similar optometry disc findings on referral in group B. Thus in the latter half of the study more appropriate patients were being referred to HES with less false positive referrals. A caveat to our findings are that by virtue of attaching optic disc images, optometry chose not to make accurate comment on disc findings in more patients in group B. While disc images are invaluable the continued practice of clinical disc analysis needs maintained to retain important clinical skills in the community and in particular documentation of disc findings in the referral even if there is an attached image.

Visual field examination took place between 81 and 84 % and this is probably a reasonable figure given that this was possible in 94 and 95 % of patients in the HES. Again there are limitations in the community with regard to equipment and patient compliance in comparison to hospital. In the future however it is to be encouraged that optometrists should strive for all patients to have computerised perimetry. The exceptions should only be patients who genuinely have difficulty with the test or those who have advanced disease whose referral should not be held back for perimetry reasons. Some patients in the hospital did not have perimetry due to physical inabilities and compliance problems. Direct comparison between optometry and HES visual fields was not possible due to the different test strategies and multiple fields.

It is most encouraging that in HES there were significantly fewer ‘normals’, more glaucoma suspects, ocular hypertensives and patients with chronic open angle glaucoma in group B than in group A. This implies that the appropriate patients were being seen in HES with less crowding by false positive referrals. This is quite a key improvement in delivering glaucoma services. Reports have suggested that the “flooding” with false positive referrals that occurred within the English NHS with the publication of NICE led to other patients within the system going blind because of delayed return appointments [22, 23]. This phenomenon was not experienced by our region and furthermore coincidentally at this time we reported on reducing numbers of blind registration as a consequence of glaucoma [15].

Comparable results were found by Ang et al. [20] They found that following the introduction of the new GOS contract in Grampian there was a significant increase in the true-positive referrals from 18 to 31.7 % with reduction of the false positive referrals from 36.6 to 31.7 %.

Our study is not without its limitations. Reliable visual fields could not always be performed at first visit and therefore patients were brought back for repeat testing. A definitive diagnosis therefore was not possible at first visit in the more obscure presentations. Low tension glaucoma in most cases was only diagnosed after phasing of IOPs and this was also not possible at first visit. Primary angle closure and primary angle closure glaucoma was also not stated as a diagnosis till additional tests and laser treatment was given at a second appointment. These patients were all categorised under “other diagnosis”. While this remains a limitation of our study we still feel that our overall patient numbers and clinical findings are significant and allude to earlier detection of glaucoma as a result of improved screening and referral processes.

There remains wider debate out with Scotland on the role of community optometry screening for glaucoma. A recent personal view from England suggested that optometry had a “scatter gun” approach to applying test strategies in the community which was unhelpful to HES [24]. However increasingly England is investing in discrete schemes that heavily collaborate with community optometrists [5, 6, 17]. A key difference is that all Scottish optometrists receive higher remuneration for a more complete ophthalmic examination, including repeat testing compared to their English counterparts. This should allow for more consistent practice across Scotland.

There is also debate on the value of free eye tests in Scotland since the new GOS contract was introduced in 2006 [25]. Statistics show that the number of eye tests conducted have more than doubled in Scotland since 2006, although with greater disparity with less usage in the lower socioeconomic groups. The economic evaluation is difficult and would have to balance the cost of free eye tests against the cost of missing potentially blinding disease and maintaining visually impaired patients in the community. Notwithstanding this, the current study shows that glaucoma patients appear to be referred earlier in the disease process with fewer false negative referrals and this is a mark of clear progress for glaucoma management in Scotland.

**Fig. 1:**
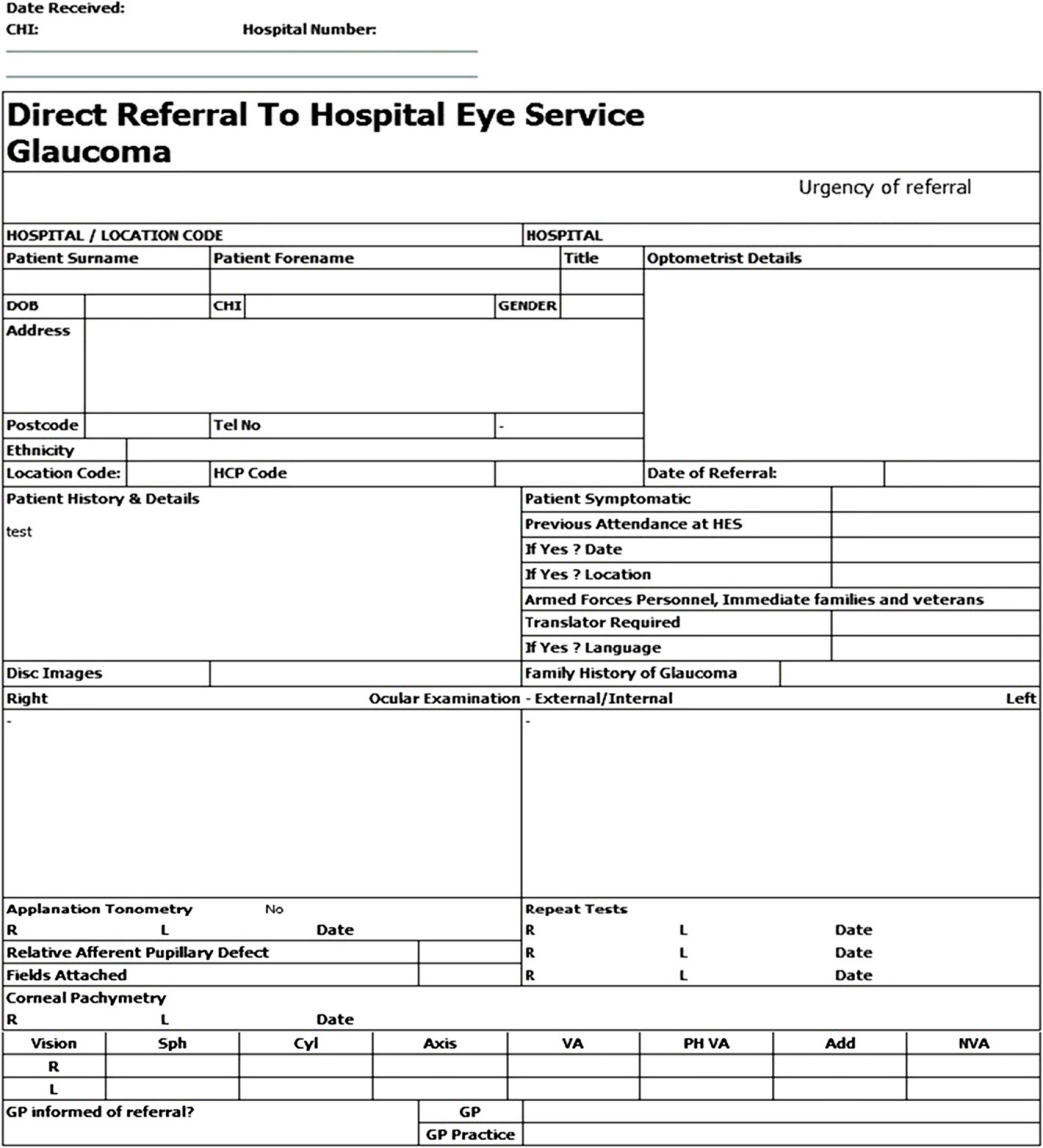
Glaucoma direct referral form to hospital eye services

The Scottish Intercollegiate Guidelines Network (SIGN) guidance for glaucoma was published in March 2015 and explicitly states the need for all three glaucoma tests before referral from community to HES. It also addresses patient criteria for hospital referral and identifies groups of patients that can be safely followed up in community [26]. The Eyecare Integration programme is being rolled out across Scotland [10] and has a glaucoma specific referral form that outlines all key clinical referral information for comprehensive screening (Fig. 1).

## Conclusion

Our study shows that patients are referred earlier in the glaucomatous disease process with fewer false positive referrals after the introduction of the GOS and EIP pilot. NICE guidelines have provided clinical guidance without increasing the number of referrals. However we also highlight that full implementation of the GOS contract is yet to be fulfilled. This goal maybe further achieved by full implementation of the EIP and SIGN guidelines for glaucoma. Further study in the next six years will hopefully show continued improvement.

## Abbreviations

EIP: Eyecare integration programme
GOS: General ophthalmic services
GP: General practitioner
GEPR: Glaucoma electronic patient record
HES: Hospital eye services
IOP: Intraocular pressure
NICE: The National Institute for Health and Care Excellence
